# Exploring Space Management Goals in Institutional Care Facilities in China

**DOI:** 10.1155/2017/6307976

**Published:** 2017-08-01

**Authors:** Lingzhi Li, Jingfeng Yuan, Yan Ning, Qiuhu Shao, Jiankun Zhang

**Affiliations:** Department of Construction Management and Real Estate, Southeast University, Nanjing 210096, China

## Abstract

Space management has been widely examined in commercial facilities, educational facilities, and hospitals but not in China's institutional care facilities. Poor spatial arrangements, such as wasted space, dysfunctionality, and environment mismanagement, are increasing; in turn, the occupancy rate is decreasing due to residential dissatisfaction. To address these problems, this paper's objective is to explore the space management goals (SMGs) in institutional care facilities in China. Systematic literature analysis was adopted to set SMGs' principles, to identify nine theoretical SMGs, and to develop the conceptual model of SMGs for institutional care facilities. A total of 19 intensive interviews were conducted with stakeholders in seven institutional care facilities to collect data for qualitative analysis. The qualitative evidence was analyzed through open coding, axial coding, and selective coding. As a result, six major categories as well as their interrelationships were put forward to visualize the path diagram for exploring SMGs in China's institutional care facilities. Furthermore, seven expected SMGs that were explored from qualitative evidence were confirmed as China's SMGs in institutional care facilities by a validation test. Finally, a gap analysis among theoretical SMGs and China's SMGs provided recommendations for implementing space management in China's institutional care facilities.

## 1. Introduction

Space management is considered an important segment of facility management [1]. Space management practices focused on optimizing the use of the existing space and reducing the maintenance operations cost [2]. Best et al. also explain that effective space management is a major source of value optimization because space is a very expensive and scarce resource in organizations [3]. Ibrahim et al. conclude many aspects that space management covers such as space strategy establishment, space planning, space environment management, and space utilization auditing [2]. As a result, space management has been practiced in several industries including educational facilities, healthcare facilities, and commercial facilities [4, 5]. However, the study of space management in institutional care facilities is limited [6].

The National Bureau of Statistics of China reports that the population aged 65 years and over in China was about 138 million in 2014 [7], and meanwhile, the China Industry Information Center reports that the total quantity of beds in institutional care facilities in 2014 had grown to approximately 5,514,000 [8]. However, the vacancy rate is approximately 60.8%, indicating an extremely poor space management performance in China's institutional care facilities. Some problems in China's institutional care facilities, such as poor living environment and space dysfunctionality, were also investigated by researchers [9, 10]. Leung et al. and Andersson et al. [6, 11] assert that the quality of the living environment is another critical factor that directly influences staff productivity and the satisfaction of elderly residents. Therefore, it appears urgent to examine space management in China's institutional care facilities.

Among major space management studies are usability assessment, process design, and performance measurement. Andersson et al. [11] used participant observation to study the space usability in assisted living facilities; Hassanain and Moied [1] developed the space management process in corporate organizations; Leung et al. and Lavy et al. [6, 12] listed space utilization and space occupancy cost as indicators to assess building performance. However, most prior researchers ignored the significance of setting goals for space management practice. In reality, setting goals (objectives) is the first step to conduct management, and the theory of goal setting illustrates that high-quality management is provided via appropriate goal setting [13]. Goals for space management are the baseline for space management practice in the process of determining which direction the facility managers and space managers should concentrate on [14]. Therefore, the need for identifying space management goals (SMGs) to guide space management practice for institutional care facilities in China is extremely clear, paramount, and urgent.

The remainder of this paper is organized as follows.  Section 2 defines and discusses the concept of space management.  Section 3 presents a literature review on space management aspects in institutional care facilities and identifies the knowledge gaps for space management goals in China's institutional care facilities.  Section 4 describes the multimethod research procedure with four steps: systematic literature analysis, intensive interviewing, qualitative analysis, and validation test.  Section 5 presents the final results obtained from qualitative analysis and validation results, which illustrates the appropriate SMGs for institutional care facilities in China.  Section 6 discusses the gap between the theoretical SMGs and China's SMGs in institutional care facilities and lists the limitations of this research.  Section 7 provides conclusive remarks and recommendations for future study.

## 2. Space Management Concept

Nowadays, space management is one of the key components in facility management. More research discussed space management practice in educational facilities, healthcare facilities, and commercial facilities [4, 5]. However, a clear and comprehensive definition of space management remains lacking. For instance, prior studies on educational facilities define space management as a process to evaluate space utilization, calculate space cost, assess space needs, and manage space changes with benchmarking tools [15, 16]. However, much research in healthcare facilities has considered space management as a process to optimize space layout and space utilization and to reduce operation and maintenance costs [17, 18]. Similarly, the FM System ARCHIBUS also states that space management manages space effectively to mitigate the cost of wasted space and to optimize the usage of space [19]. In addition, the research on workplace management indicates that space management can support core business goals and meet users' requirements [20, 21]. However, this description does not show how to support business goals through space management. Furthermore, the chapter on space planning and management listed by Roper and Payant illustrates that space management is a highly dynamic process due to the interactions between space, users, activities, and technologies [22].

Based on the analysis noted above, this paper defines space management as an interdisciplinary endeavor that incorporates space, users, activities, and technologies to plan and manage a working/living environment that effectively supports core business goals. Several variables like space utilization, space occupancy cost, space flexibility, and space accessibility should be balanced to achieve effective space management through the whole process of planning and managing the working/living environment [20]. In general, space management is a tool that can be leveraged to support core business goals, such as revenue growth and profitability growth [23]. It is notable that the core business goals could be changeable according to facility types and organizational culture.

## 3. Related Works

In order to explore space management goals in institutional care facilities in China, the literature review on space management components in institutional care facilities, the goal setting aspects of space management, and the general research methods are necessary. The following subsections which describe the corresponding review results can be helpful in determining the research content and the research approach.

### 3.1. Literature on Space Management Components in Institutional Care Facilities

Institutional care facilities provide accommodation and rehabilitative, restorative, and/or ongoing skilled nursing care to the elderly who are in need of assistance with their daily living activities [24, 25]. Because elderly care is organized differently in countries or regions, the terminology of institutional care facility may differ accordingly. The institutional care facilities in Mainland China mainly include senior apartments, residential care homes, and nursing homes [26]. And institutional care facilities are also called care and attention (C&A) homes in Hong Kong, assisted living facilities and retirement homes in America and in Sweden, and rehabilitation facilities in Japan [27, 28]. Due to the similar environments, relevant research on the above different types of facilities are partly applicable to institutional care facilities in China [11]. In such facilities, prior studies [6] illustrated that improving the quality of the physical environment provides a comfortable and healthy environment for elderly residents and also care staff. Therefore, many prior researchers into architecture design and building performance assessment about institutional care facilities have been conducted. Leung et al. [4] defined the space-related components of facility management as architecture aspects that concern the layout and design of C&A homes in Hong Kong for the purpose of enhancing their environmental qualities and the health status of elderly residents. Preiser [29] stated that the postoccupancy evaluation (POE) method to evaluate space performance from users' perspective was first noted in institutional care facilities. Meanwhile, certain space-related problems were identified including health and safety, way-finding problems, environmental issues, space accessibility, and assignment problems [29]. Andersson et al. [11] studied the daily use of common spaces in assisted living facilities and showed several physical environment problems such as conflicts of space use and poor accessibility. Zhou and Chen [10] proposed some design suggestions of aged care facilities to save operation cost and improve the environment safety.

The aforementioned architectural aspects including optimizing space layout and managing physical environment can be regarded as space management components. Besides that, other typical management components or functions, such as auditing space utilization and charging space occupancy cost, are also effective measures to conduct space management [2]. The performance of these typical components has been illustrated in educational facilities and commercial facilities. For instance, Ibrahim et al. [2] developed a space charging model to optimize space use and minimize operation cost in higher education institutions; Hassanain and Moied [1] designed the auditing process to improve space utilization rate in corporate organizations. However, very few research studies have proposed those typical space management functions in institutional care facilities [4, 11]. To some extent, those typical management aspects represent the efficiency of space use while architectural aspects mostly represent the functionality or effectiveness of space use. In other words, those architectural aspects and management aspects can illustrate the goals that space management could be achieved. The integration of all space management components or functions can develop comprehensive goals for space management in institutional care facilities. Therefore, these architectural aspects as well as typical management components should be noted in searching for space management goals in institutional care facilities.

### 3.2. Literature on Goal Setting for Space Management

The theory of goal setting clearly described the significance of performance improvement by setting management goals [13]. However, prior studies related to space/workplace management, facility (asset) management, and building performance focused minimally on setting goals for space management [4, 28]. Regarding the goal setting process, Campbell and Finch [30] proposed that the process of establishing goals in the field of facilities management was not straightforward, since different stakeholders tended to meet their own facilities management requirements and also there is no applicable principles to identify goals. Therefore, it is also challenging to set space management goals for China's institutional care facilities.

According to the definition of space management, supporting core business goals is the ultimate objective of space management. Lindholm and Leväinen [23] stated two strategic core objectives in corporate organizations including revenue growth and profitability growth. In institutional care facilities, the satisfaction of elderly residents that can directly reflect the quality of life was emphasized by many researchers [6, 31]. Thus, improving users' satisfaction should be confirmed as one of the core business goals in institutional care facilities. All these three core business goals can provide strategic directions for exploring comprehensive space management goals in institutional care facilities.

One effective way to identify management goals is the literature review approach, which emphasizes the creation of new knowledge through referring to existing research [32]. This approach is gaining wider acceptance in many prior studies. For example, Yuan et al. [33] used the literature review approach to select the best value and then transferred the best value indicators to performance objectives. Based on extensive literature, Lavy et al. [32] identified the key performance indicators of facility management to determine the process towards building performance goals. Semistructured interviews is another approach to select goals, in which new ideas will be brought up during the interviewing process [34]. Specifically, Ohura et al. [34] conducted semistructured interviews to identify the care goals in facilities for elderly people. To determine the environment objectives, Nousiainen and Junnila [35] conducted semistructured interviews to identify end-user requirements for green facility management. In terms of methods, all these studies disintegrated the theoretical approach and the practical approach, which might result in a gap between theory and practice. Applying mixing methods between literature review and interview approach to explore space management goals in institutional care facilities in China would be more scientific to recognize the appropriate goals for China's space management practice and meanwhile explore the potentially theoretical goals that be needed in China's institutional care facilities in future.

### 3.3. Knowledge Gaps

The above literature review results present three knowledge gaps for space management goals in institutional care facilities in China. First, typical space management components that can represent efficiency goals for space management have not been practiced in institutional care facilities. Second, the principles of space management goals were not clearly proposed, which would increase the difficulty of setting space management goals for institutional care facilities in China. Furthermore, most research for identifying management goals resulted in a gap between theory and practice. To fill these knowledge gaps, this study's objective is to explore space management goals (SMGs) for institutional care facilities in China through careful review of the literature and critical observation of practices in China's institutional care facilities.

## 4. Research Methodology

**Figure 1:**
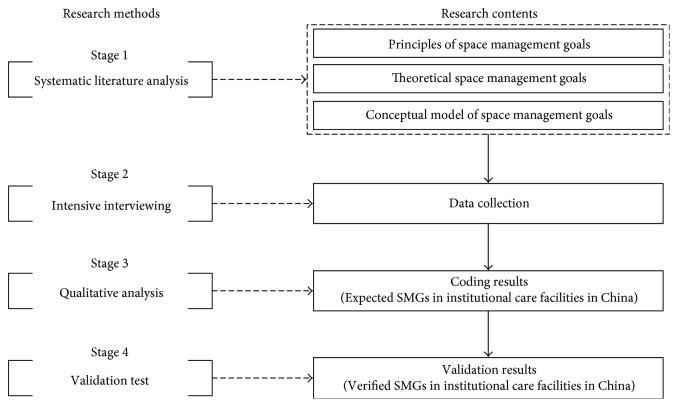
The research procedure to explore SMGs in institutional care facilities in China.

Systematic literature analysis, intensive interviews, and qualitative analysis approach have been frequently used to select paramount determinants of effective space management or to develop new theories [20, 36]. Combining those approaches can be an effective methodology to explore space management goals in institutional care facilities in China. The research procedure of this study includes research methods and research contents, as presented in Figure 1. This procedure is a step-by-step methodology consisting of four stages.

In the first stage (stage 1), principles of SMGs, theoretical SMGs in institutional care facilities, and the conceptual model of SMGs are developed through systematic literature analysis. Data collection through intensive interviewing is conducted in the second stage (stage 2). The intensive interviewing, which collects stakeholders' views about SMGs, is a useful data-gathering approach that matches the qualitative method precisely. Prior studies indicated that goals should represent stakeholders' expectations and requirements [33, 37]. In this context, the stakeholders include facility owners, facility managers, care staff, elderly residents, and their families. In the third stage (stage 3), the qualitative analysis processes, including open coding, axial coding, and selective coding, are performed to select expected SMGs for institutional care facilities in China. In the last stage (stage 4), the conceptual model of SMGs will be as the validation tool to verify SMGs in institutional care facilities in China. The first three research methods are relatively complex, which are further described as follows.

### 4.1. Systematic Literature Analysis

**Figure 2:**
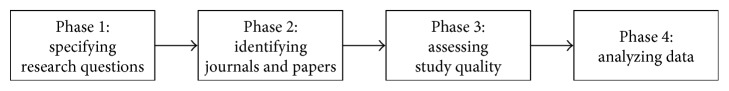
Systematic literature analysis process.

Performing a systematic literature analysis involves several activities [38], which can be grouped into four main phases (see Figure 2).

In the first phase, regarding the topic of exploring SMGs for institutional care facilities, the following two research questions are in focus: (1) what are principles that SMGs should satisfy? (2) What are the theoretical SMGs can be suitable for institutional care facilities?

**Figure 3:**
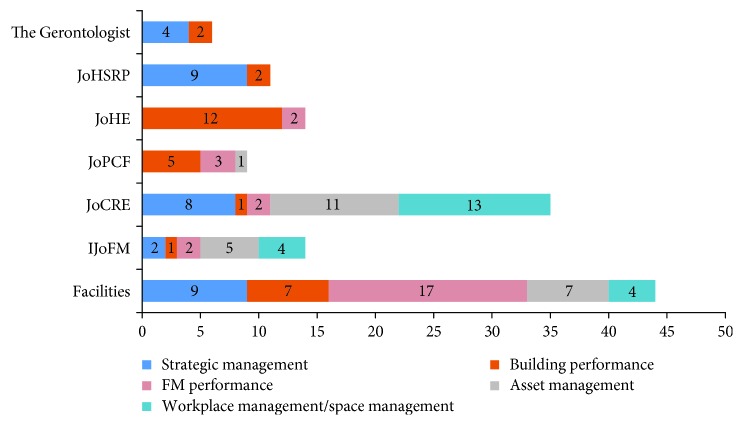
Distribution of reviewed paper by journals and searching keywords.

The second activity is to identify journals and papers that are likely to cover the above research questions. Here, four top FM-focused journals and three journals related to elderly care facilities were recommended from FM researchers and Gerontology researchers. These journals include Facilities, Journal of Facilities Management (JoFM), Journal of Corporate Real Estate (JoCRE), Journal of Performance of Constructed Facilities (JoPCF), Journal of Housing for the Elderly (JoHE), Journal of Health Services Research & Policy (JoHSRP), and The Gerontologist. Meanwhile, five keywords are explored based on research topics and questions including *strategic management goals*, *building performance*, *facility management performance*, *asset management*, and *workplace management and space management*. The following criteria are used to select studies in the present review: (1) focus on space management/facility management or at least mention space-related performance indicators and (2) list of space problems from architecture aspects for institutional care facilities. Searching with keywords and applying these selection criteria, a total of 133 articles from 1998 to 2016 have been selected from the above journals. All these selected articles were sorted into five categories according to the searching keywords (Figure 3).

Prior studies use various dimensions to perform quality assessment, such as size of study, methodology setting, theoretical basis of the study, and statistical methods [39]. Based on that, this paper chooses three criteria to assess the quality of selected articles in the third phase. These criteria include a clear description of study setting, whether the content is pertinent to space management, and whether the space management aspects can be generalized to institutional care facilities. A study is considered of high quality if it meets these 3 criteria. Any disagreements between coresearchers were resolved by discussion. After application of these quality assessment criteria, 86 high-quality articles were selected for data analysis.

The fourth phase describes the results of data analysis of selected high-quality articles, which is based on the research questions that were given in phase 1. After checking the content of each high-quality article and the space management concept, two principals were set to identify SMGs for institutional care facilities. At the strategic level, SMGs should support the organizational core business goals, that is, principle 1: adding value to core business [40, 41]. In institutional care facilities, core business goals include revenue growth, profitability growth, and improvement in elderly residents' satisfaction levels [31, 42]. At the operational level, the theory of goal setting illustrates that SMGs should enhance the space-related performance [13]; this is set as principle 2. The term “performance” is related to the efficiency and effectiveness of space use [43]. Andersson et al. also explained that, in the context of assisted living facilities, efficiency is associated with the spent space resource and the effectiveness related to the desired space function [11]. According to abovementioned two principles, the space-related problems and the performance indicators can be transferred into theoretical SMGs for institutional care facilities. Finally, the systematic literature analysis process identified 9 theoretical SMGs for institutional care facilities from these selected high-quality articles (see Table 1).

**Figure 4:**
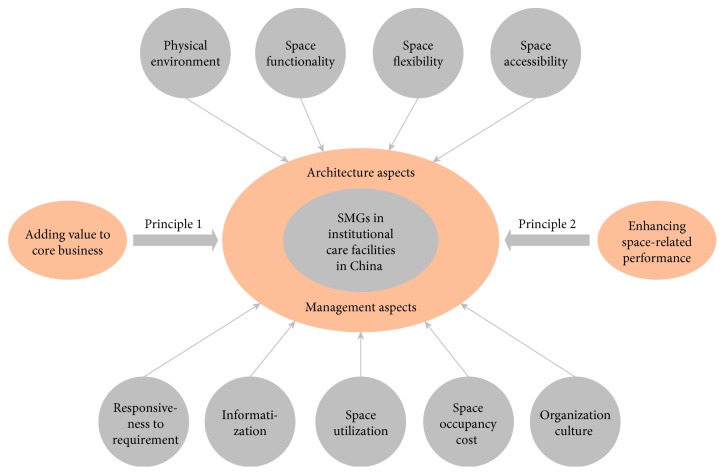
Conceptual model for exploring SMGs in institutional care facilities.

In addition, the selected 9 theoretical SMGs (see Table 1) as well as two principles of SMGs are combined to develop a conceptual model of space management goals for institutional care facilities (Figure 4()). The selected theoretical SMGs include architecture aspects (physical environment, space functionality, space flexibility, and space accessibility) and management aspects (responsiveness to requirement, informatization, space utilization, space occupancy cost, and organization culture). Besides, principles 1 and 2 in this model can be filter conditions to verify the SMGs that explored from institutional care facilities in China. Crucially, this conceptual model of space management goals provides the clues for designing intensive interview guidelines (see Table 2), which can be thought as the guideline for data collection.

### 4.2. Intensive Interviewing

To collect data from the in-practice perspective of stakeholders' opinions, seven facilities including three residential care homes and four nursing homes were visited and interviews were conducted with intensive interview guidelines (see Table 2). These facilities are spread throughout China (2 in Beijing, 2 in Shanghai, 2 in Nanjing, and 1 in Nanchang). Specifically, these facilities are private organizations; this type represents the current largest segment of institutional care facilities in China.

Collecting data by intensive interviewing involves two steps. The first step is initial sampling, which involves conducting intensive interviews in the first facility to find relevant materials and directions for the core category [54]. The core category was defined as “expected SMGs” in this study. Thereafter, the second step is theoretical sampling, which intends to elaborate and refine theoretical categories and to specify the relations among categories for the axial coding process [54]. In addition, the theoretical sampling was halted in the fifth facility since there were no new categories related to “expected SMGs” that could be obtained from facility 6 and facility 7. This finding means that theoretical saturation was achieved and the data collection process for the qualitative analysis can be completed [54].

The participants in the interviews were 6 facility owners, 1 facility manager, 6 care staff, 4 elderly residents, and 2 elderly residents' family members; these individuals represent all stakeholders' opinions in this research. All data were collected from April 2016 to August 2016, among which only facility 5 in Beijing was interviewed through email; the other facilities were surveyed on site. The background information of the seven facilities that were interviewed is presented in Table 3.

### 4.3. Qualitative Analysis

The qualitative analysis, an inductive approach, is conducted to explore “expected SMGs” in institutional care facilities in China through open coding, axial coding, and selective coding. First, the coding process required identifying and conceptualizing the overall contents as free nodes that are relevant to SMGs from the collected intensive interview data [55]. Second, axial coding is used to compare categories with the collected free nodes and relate categories to subcategories [56]. The relationship between categories and subcategories was called a “parent-child” relationship in this research. Through iterative, inductive, and deductive analysis, parent nodes, child nodes, and major categories will be identified in axial coding process. Finally, the selective coding process specified the multiple relationships among the categories [56]. A single storyline to locate and explain the most salient aspects of major categories and their multiple relationships will be developed in this paper. Meanwhile, the final edition of expected SMGs in institutional care facilities in China is explored.

## 5. Results

The computer software package named NVivo version 11 (QSR International) was employed to assist in conducting three coding processes in qualitative analysis [57]. The coding results from qualitative analysis and the validation results from validation rest were clearly described in the following subsections.

### 5.1. Open Coding

**Figure 5:**
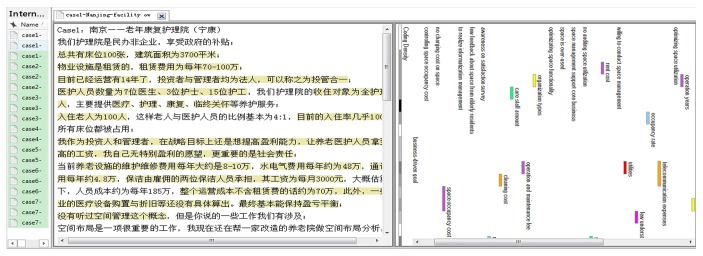
Open coding of the interview materials for exploring SMGs in institutional care facilities in China (facility 1—Nanjing—facility owner).

Due to the length limitation of this paper, the open coding results for all materials could not be extended. Only one interview result (Facility 1-Nanjing-Facility Owner) was presented as an example to show the open coding results; this is shown in Figure 5. The information on the left in Chinese regards the interview material with the coded words or sentences. The information on the right with diverse colors regards the coding stripes as well as the free notes. Free nodes were the initial nonhierarchical categories and the corresponding conceptions. Consequently, a total of 77 free nodes were open coded, which assisted with the axial coding and selective coding.

### 5.2. Axial Coding

**Figure 6:**
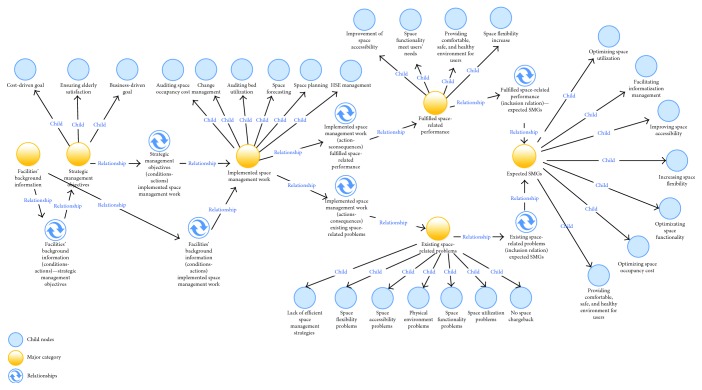
Concept map of exploring SMGs through the qualitative analysis approach.

After categorization, the selected 77 free nodes were grouped into 8 parent nodes and 83 child nodes.  Table 4 illustrates the parent nodes and only a portion of the child nodes due to the length limitation in this paper. Most of the child nodes are shown in Figure 6. Through the initial analysis of the correlation between the categories and the core category (expected SMGs), this study selected six major categories including facilities' background information, strategic management objectives, implemented space management work, existing space problems, fulfilled space-related performance, and expected SMGs. Consequently, there were 19 space-related problems identified in this coding process. The corresponding consequences and sources are also shown in Table 5. Most of these space-related problems have a direct negative effect on the organizational core objectives such as a decrease in profitability, staff productivity, and elderly residents' satisfaction.

### 5.3. Selective Coding

Through the content analysis of the interview results, there were four relationships among categories. The first link was the “parent-child” relationship that was marked in the axial coding process. Second, the relationship between strategic management objectives and the organizations' background information to perform space management work was called the “conditions-actions” relationship. Using facility 1 as an example, there was a surplus of care staff in this nursing home, and the bed utilization rate was nearly 100%. In addition, there were increasingly more clients waiting for beds. The facility owner stated that the strategic management objective of this nursing home was to increase revenue; therefore, the facility owner planned to enlarge the facility's scale in the future. Consequently, space forecasting and planning were performed.

The third relationship is referred to as an “actions-consequences” relationship. Through the existing space management actions, certain space-related performance measures were fulfilled including providing a comfortable, safe, and healthy environment, optimizing space functionality, and improving space flexibility and space accessibility. Furthermore, certain space-related problems were also proposed by interviewees as negative consequences.

Finally, regarding the inclusion relation as the fourth relationship, both the fulfilled space-related performance and the existing space-related problems could be transferred to “expected SMGs” as the stakeholders' desired outcome achieved by implementing space management. Based on the multiple relationships among the major categories, the concept map inside NVivo version 11 (QSR International) was adopted to visualize the path diagram of exploring SMGs in institutional care facilities in China (Figure 5). Most importantly, the child nodes of expected SMGs in Figure 5 were the final “expected SMGs” in institutional care facilities in China.

### 5.4. Validation Results

The conceptual model of space management goals (Figure 4) was used as the validation tool to verify whether the selected “expected SMGs” could be confirmed as SMGs. To perform this validation, stakeholders' opinions were incorporated into the validation matrix (Table 6); principles being satisfied in each space management goal were marked “**√**”; this illustrated that each expected SMG satisfied both principle 1 and principle 2. Therefore, all expected SMGs were confirmed as SMGs for institutional care facilities in China. In addition, most of these expected SMGs were consistent with their corresponding theoretical SMGs. This consistency further validated the conceptual model of SMGs for institutional care facilities.

## 6. Discussion

### 6.1. Gaps between Theoretical SMGs and China's SMGs

**Figure 7:**
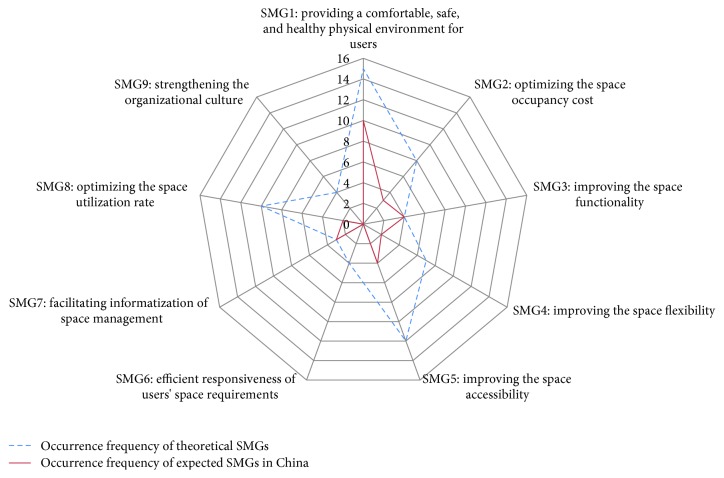
Occurrence frequency of each theoretical SMG and China's SMG.

The data analysis and results reveal that there is a gap between the theoretical SMGs and China's SMGs in institutional care facilities. This gap includes quantity differences and content differences. To illustrate the quantity differences, our research defined the number of selected references for each theoretical SMGs (Table 1) and the number of sources for each expected SMGs (Table 4) as the “occurrence frequency,” and then compared the occurrence frequency for each SMGs (Figure 7). Moreover, content differences were explored using content analysis. Consequently, several comparison results are discussed further below.

1. A lack of corresponding demand and awareness leads to a low efficient responsiveness of space management and development of organizational culture in China's institutional care facilities.

In prior research, SMG6 and SMG9 were explored. Hinks and McNay selected the goal of SMG6 as the indicator for determining residential satisfaction and evaluating staff productivity [51]. However, facility owners and facility managers did not propose SMG6 and did not use it as the customer satisfaction indicator. Interviewees explained that elderly residents, particularly the elderly lying in bed, seldom proposed space-related requirements. In addition, the goal of strengthening the organizational culture through space management (SMG9) was also neglected by interviewees, since they did not notice that space is the medium for expressing organizational culture and values. However, this goal was of more concern due to its benefits to the corporate culture and to the core business brand in recent workplace studies [53].

(2) The elderly daily living and organizational care business strongly depend on a high-level environment, accessibility, and functionality for China's institutional care facilities.

Providing a comfortable, safe, and healthy physical environment (SMG1) and improving the space accessibility (SMG5) were more significant in the institutional care facilities observed that were open 24 hours per day, 7 days per week and that provided a living environment for elderly residents with physical disabilities. These two goals were also the most significant space-related requirements that elderly residents and their family members proposed. In addition, optimizing the space functionality (SMG3) was usually focused on ensuring space function support during the whole building life cycle for the organizational care business. Therefore, both in practical facilities and in previous studies, facility owners and facility managers were strongly concerned with these three goals.

(3) Different users' preference and local conditions could hinder the improvement of space flexibility due to the disapproval of China's institutional care facilities.

To improve the space flexibility (SMG4), previous studies illustrated that creating more sharing spaces and open space to increase flexibility was conducive [20, 47]. However, facility owners in facility 3 and facility 4 stated that sharing space strategies were disapproved in several China's institutional care facilities because the multiple functions of this aspect of space flexibility confused elderly residents. The facility owner in facility 4 also proposed that a sharing space strategy should be implemented based on users' experience. Furthermore, SMG4 with an open-planning strategy could help organizations respond to business changes quickly and reduce costs. A negative example depicted that the facility owner in facility 3 complained of the difficulty of changing the space from a dependent layout to an independent layout due to the lack of open space.

(4) Auditing interior space, space chargebacks, and informatization management would be helpful in optimizing the space occupancy costs and improving the space utilization rate.

Regarding optimizing the space occupancy cost (SMG2), most of the facilities observed proposed that the task of auditing space occupancy cost be performed by the financial department. In addition, FM managers did not focus more on these financial data or charge the cost to care units (space). However, space chargeback was the most popular strategy to optimize costs in the FM research [2]. Only the facility manager in facility 3 stated auditing the space occupancy cost as well as the strategy of space chargebacks would be applied in his nursing home because he had experience with this work in hospitals. Specifically, the space occupancy cost represented 30%–50% of the total cost in all observed facilities, which was often regarded as the second highest expense after personnel costs. Hence, appropriately optimizing the space occupancy cost in these observed facilities could lead to large benefits for facility owners, particularly for most facilities that could only achieve the break-even point or be under deficit.

The requirement of informatization systems (SMG7) in the institutional care facilities observed was for bed management and financial management, not for the classic space management work such as space utilization and space occupancy cost auditing; this needs to be explained further. Regarding space utilization auditing, interviewees believed this referred to auditing the bed occupancy rate, instead of the prior research that highlighted that auditing was the space use rate for all interior space such as common space, living space, communication space, and staff workplace. Therefore, there was no space utilization rate data in the observed facilities. It was anticipated that several interviewees would propose requirements to optimize the space occupancy cost (SMG2) and the space utilization rate (SMG8) once they understood the contents of space management after the intensive interviews. In China's institutional care facilities, these two goals were difficult to achieve since the work of auditing the space utilization rate and the space occupancy cost has not been performed yet.

(5) SMGs could provide different priorities to fit the changes in the organizational environment and the stakeholders' requirements.

In practice, SMGs will vary or evolve over time in addition to the changes of facility scale, elderly care services, and management awareness. For example, the facility owner in facility 7 stated that SMG7 was not necessary in his nursing home (80 beds). Then, the owner explained that staff could conveniently manage beds and rooms with Microsoft Excel and update data manually in small facilities; in addition, the informatization systems could not deliver efficiency to the space management work in small facilities. Furthermore, although SMG6 and SMG9 were not currently accepted by China's institutional care facilities, these goals could be added when facility owners or facility managers understand the added value of these SMGs and when they know the systematic process of space management in institutional care facilities.

### 6.2. Research Limitations

Although this study explored SMGs in institutional care facilities from both theoretic and practical perspectives, two limitations should be more concerned. The first limitation is the fact that data was only collected from private facilities without considering public facilities. Space management goals may not be the same in different operation types of institutional care facilities; such as, optimizing the space utilization rate (SMG8) should be more concerned in private facilities than in public facilities. Correspondingly, these explored SMGs this research should be further verified in the future through interviewing public institutional care facilities. Second, the added value of improving the efficiency and effectiveness of space use as well as supporting core business goals that space management brings was only illustrated by interviewees' descriptions in a qualitative way. However, a quantitative analysis will be needed to illustrate the added value of space management clearly in future study.

## 7. Conclusion

Prior research on China's practice in institutional care facilities denote an absence of goals for space management, which means practitioners lack a specified direction to implement space management in China. To explore space management goals (SMGs) in institutional care facilities in China, this paper has conducted a multimethod research strategy.

Through systematic literature analysis, two principles for identifying SMGs were set; nine SMGs were subsequently selected as theoretical SMGs in institutional care facilities; a conceptual model of SMGs in institutional care facilities was developed to guide the following intensive interviewing process. Thereafter, the qualitative analysis has revealed 7 SMGs by investigating 7 institutional care facilities in China. During the analysis of the interview results using three coding processes, six major categories, as well as their interrelationships were identified to visualize the path diagram of exploring expected SMGs in institutional care facilities in China. Furthermore, 19 space-related problems as well as their corresponding negative consequences were identified in the 7 institutional care facilities observed. In addition, all expected SMGs were confirmed as SMGs for institutional care facilities in China through a validation test.

To conclude, the comparative analysis of 9 theoretical SMGs and 7 China's SMGs provides the following recommendations that can be adopted to implement space management in practice: (1) training FM staff to identify the added value of space management and to know how to conduct space management systematically is urgently needed; (2) more efforts should be contributed to improving the physical environment, the space accessibility, and the space functionality; (3) open-space strategies can be practiced to improve the space flexibility in China's institutional care facilities; (4) an audit of the interior space, space chargebacks, and informatization management should be conducive to the optimization of the space occupancy cost and the space utilization rate; and (5) SMGs should be prioritized according to the organizational environment and the stakeholders' requirements.

In view of the results and research limitations, future studies should focus on validating whether these explored SMGs can be applicable in public institutional care facilities in China. For a wide application, space management goals may need further adjustment. To clearly illustrate the added value of space management for institutional care facilities, quantization criteria and assessment should be developed in future work.

## Figures and Tables

**Table 1:** Theoretical space management goals from the literature review.

Theoretical SMGs	Descriptions	Added value to core business or space-related performance	Representative sources	Number of references
SMG 1: providing a comfortable, safe, and healthy environment for users	Includes warm-style design, good ventilation, comfortable lighting, safety layout, clean environment, and effective noise control.	Good indoor physical environment has positive impacts on the quality of elderly care, residents' satisfaction, and staff productivity.	[31, 44, 45]	15
SMG 2: optimizing the space occupancy cost	Includes space rent or building depreciation and reconstruction costs, utility costs, housekeeping costs, repair and maintenance costs, HSE costs, and moving costs.	This goal is vital to organizational profitability growth since space occupancy costs are usually the second largest component of total cost in organizations.	[20, 31]	8
SMG 3: optimizing the space functionality	Strives to ensure that each space fulfils the functions of its intended use, such as sufficient space and critical function for various intended operations and users' requirements.	This goal's objective is to support users' space requirements and the organizational business processes.	[11, 29]	4
SMG 4: improving the space flexibility	Requires the building to accommodate frequent alteration, renovation, and multiple use quickly and economically. Strategies include space sharing and open-space design.	This goal can enhance organizational profitability through quickly responding to business changes, reducing renovation costs, increasing the space utilization rate, and improving staff productivity.	[23, 46–48]	7
SMG 5: improving the space accessibility	Involves person-environment interaction that includes barrier-free environment, alternative orientation systems, minimizing circulation distances, and efficient work flows and logistics.	This goal directly increases elderly residents' satisfaction and staff productivity.	[11, 49, 50]	12
SMG 6: efficient responsiveness to users' space requirements	Strives to ensure the FM department can respond to space-related problems or users' space-related requirements efficiently.	This goal can increase the elderly residents' satisfaction and can be an indicator to evaluate staff productivity.	[41, 51]	4
SMG 7: facilitating informatization of space management	Utilizes building information to perform space planning, space inventory, and cost charges through IT tools.	This goal can add value to organizational profitability through optimizing space use resources and reducing personal costs.	[50, 51]	3
SMG 8: optimizing the space utilization rate	Optimizes the efficiency of space use on the premise of end-user satisfaction. This rate is determined by the occupancy area and the occupancy time.	This goal ensures elderly residents' satisfaction and the efficient use of space resources.	[12, 20]	10
SMG 9: strengthening the organizational culture	Uses space management to enhance the business brand and to strengthen the organizational culture since space is a medium for expressing organizational culture and values.	This goal adds value to staff productivity and elderly residents' satisfaction.	[52, 53]	4

**Table 2:** Interview guide to explore SMGs in China's institutional care facilities.

Semi-structured interview questions	Interviewees
*Background information*	
Q1. What is the scale of this facility (including beds and square meter)?	Facility owner/manager
Q2. How did you acquire this property? Is it rented or owned? Can you tell us whether there a rent fee or a property depreciation fee?	Facility owner/manager
Q3. How long has this facility been operating?	Facility owner/manager
Q4. How many care-staff do you have? What kind of care do you supply?	Facility owner/manager
Q5. What is the occupancy rate for each care type?	Facility owner/manager
Q6. Do you set strategic management objectives for facilities? If yes, please explain these objectives, such as business-driven or cost-driven goals.	Facility owner/manager
Q7. Can you list the facility operation expenses, including the operation and maintenance fee, utilities, and HSE expenses?	Facility owner/manager
*Thoughts upon space management*	
Q8. Do you know the concept of space management? (This work includes space plans, space utilization audits, space occupancy cost audits, change management and space inventory.)	Facility owner/manager
Q9. Do you perform this space management work? If yes, please explain how to implement this work? What factors do you consider during the management implementation? Please list some existing completed space-related work.	Facility owner/manager
Q10. Do you have some space problems that could not be solved now, such as over-use or under-use, conflicting workflows and other users' complaints or any blocking factors for your business?	Facility owner/manager
Q11. Do you know how much space you have and how much space you will need in the future? Do you think the space is utilized efficiently?	Facility owner/manager
Q12. Do you know what is the cost per square meter in this facility? Or the facility operation cost per unit? Is the indicator of the cost being used for performance evaluation in your facility?	Facility owner/manager
Q13. Do you set indicators about the space environment in the satisfaction survey? If yes, what is the satisfaction rate for the space aspects?	Facility owner/manager
Q14. Do you consider whether space management can support your business (revenue and profitability growth, an increase in residents' satisfaction)? Are you willing to implement effective space management work?	Facility owner/manager/care staff
Q15. What kind of performance do you want to achieve through space management?	Facility owner/manager/care staff
Q16. What kind of space/environment would you desire? Do you think this facility's space satisfies your requirements? If not, please list the existing space problems.	Care staff/the elderly or their family
Q17. Please feel free to comment on any other space issues you would like to discuss.	All interviewees

**Table 3:** An overview of the observed seven China's institutional care facilities.

Facility types	Location	Elderly care services	Facility ownership	Scale	Participants' occupations
Facility 1—nursing home	Nanjing	Daily care, nursing care, rehabilitation service, and hospice care for dependent elderly residents	Rent	100 beds 3700 m^2^	Facility owner/doctor/the elderly family member
Facility 2—nursing home	Beijing	Daily care, nursing care, recreation activities, rehabilitation service, and hospice care for dependent and independent elderly residents	Rent	676 beds 40,772 m^2^	Facility manager/the elderly
Facility 3—senior apartment	Shanghai	Daily care, rehabilitation service, social worker service for dependent elderly	Rent	360 beds 10,000 m^2^	Facility owner/nurse/the elderly
Facility 4—residential care home	Shanghai	Day care, home care, daily care, health care, recreation activities for independent elderly residents	Free	500 beds 8000 m^2^	Facility owner/nurse/the elderly
Facility 5—residential care home	Beijing	Daily care, nursing care, and recreation activities for independent and dependent elderly residents	Rent	350 beds 5200 m^2^	Facility owner/nurse/the elderly family member
Facility 6—nursing home	Nanjing	Daily care, nursing care, and recreation activities and hospice care for independent and dependent elderly residents	Rent	126 beds 2600 m^2^	Facility owner/nurse/the elderly
Facility 7—nursing home	Nanchang	Daily care, nursing care, rehabilitation service, hospice care, and social services	Rent	80 beds 1800 m^2^	Facility owner

**Table 4:** Parent nodes and a part of child nodes.

Parent nodes	Child nodes	Sources
Expected SMGs	Providing a comfortable, safe and healthy environment for users	10
	Optimizing the space functionality	4
	Optimizing the space occupancy costs	3
	Facilitating informatization management	3
	Improving the space accessibility	3
	Optimizing the space utilization	2
Existing space-related problems	Shown in Table 5	16
Fulfilled space-related performance	Providing a comfortable, safe and healthy environment for users	11
	Improving the space accessibility	9
	Meeting users' requirements through space functionality	2
	Improving the space flexibility	2
Awareness on space management	Misunderstanding space management before interview	6
	Knowing the responsible staff for space management	6
	Supporting core business through space management	5
	Willing to conduct space management	5
	Receiving minimal feedback about space from elderly residents	2
	Complaining of unsystematic space management work	1
Strategic management objectives	Business-driven goal	3
	Cost-driven goal	2
	Improving elderly satisfaction	2
Facilities' background information	Elderly care types	7
	Facility scale	7
	Facility ownership	7
	Operation years	7
	Occupancy rate	7
	Profitability analysis	7
Implemented space management work	Shown in Figure 5	5
Space management strategies	Managing space to meet users' requirements	1
	Space chargeback	1
	Sharing space	2

**Table 5:** Space-related problems and corresponding consequences in the institutional care facilities observed.

Categories	Space-related problems	Consequences	Sources
Physical environment	Poor ventilation	Users' complaints and the decrease in staff productivity and elderly residents' satisfaction	Facility 3, facility 7
	No central air conditioner	Decrease in the elderly residents' satisfaction	Facility 2
Charging space occupancy cost	No auditing space occupancy cost and no charge back of space occupancy cost to space	Space occupancy cost was not decreased; this blocks a profitability increase	Facility 1–facility 7
Space functionality	Lack of rest space for staff	Decreases staff satisfaction and their productivity	Facility 1, facility 3, facility 6, facility 7
	Lack of public activities space	Decreases elderly residents' satisfaction	Facility 2, facility 6, facility 7
	Lack of storage space	Inconvenience to the staff and decreases their productivity	Facility 3, facility 6, facility 7
	One person-living room was too large for the elderly	Increase loneliness among elderly residents who live in large rooms	Facility 2
Space flexibility	Sharing public activities space	Confuse elderly residents with the multiple function use of space	Facility 3, facility 4
	Lack of open space	Difficult to renovate space	Facility 3
Space accessibility	Poor accessibility for disabled elderly residents	Decreases the satisfaction of elderly family members and decreases the productivity of care staff	Facility 3, facility 6, facility 7
	Unclear signage	Elderly residents' complaints	Facility 2
	Long communication distance for staff	Decreases staff productivity	Facility 3–facility 7
	Workflow was not fluent	Decreases staff productivity	Facility 2
	Staff has poor visibility to nursing areas	Decreases staff productivity	Facility 2
Space utilization	Space is overused	Poor flexibility and complaints about overcrowding problems	Facility 1, facility 5
	Space is underused	Waste space and increase operation cost	Facility 3, facility 5
	No auditing utilization rate for each space	Without data basis to optimize space use and to charge space occupancy cost	Facility 1–facility 7
Space management strategies	Lack of efficient space management strategies	Lower space performance	Facility 6, facility 7

**Table 6:** SMGs completed validation matrix for institutional care facilities in China.

Expected SMGs	Occurrence frequency in observed facilities	Principle 1—strategic level			Principle 2—operational level		Confirmed as SMGs
		Revenue growth	Profitability growth	Residents satisfaction increase	Space use with efficiency	Space use with effectiveness	
Providing a comfortable, safe, and healthy environment for users	10			**√**		**√**	**√**
Optimizing the space occupancy cost	3		**√**		**√**		**√**
Optimizing the space functionality	4	**√**		**√**		**√**	**√**
Improving the space flexibility	2		**√**		**√**		**√**
Improving the space accessibility	3			**√**		**√**	**√**
Facilitating informatization management	3		**√**		**√**		**√**
Optimizing the space utilization rate	2		**√**	**√**	**√**		**√**
